# Effect of Functional Water on the Antioxidant Property of Concentrated Reconstituted Juice

**DOI:** 10.3390/foods11162531

**Published:** 2022-08-21

**Authors:** Tongjiao Wu, Mitsuki Sakamoto, Natsuki Inoue, Kotaro Imahigashi, Yoshinori Kamitani

**Affiliations:** 1 The United Graduate School of Agricultural Sciences, Kagoshima University, 1-21-24 Korimoto Kagoshima, Kagoshima 890-0065, Japan; 2 Graduate School of Agricultural, Forestry and Fisheries, Kagoshima University, 1-21-24 Korimoto Kagoshima, Kagoshima 890-0065, Japan

**Keywords:** concentrated juice, alkaline electrolyzed water, tourmaline water, antioxidant activity

## Abstract

People often consume juice to easily ingest antioxidants, which can scavenge free radicals and reduce the risk of lifestyle-related diseases. In this study, the SOD assay kit-WST method was used to evaluate the antioxidant activity of two types of functional water, alkaline electrolyzed water (AlEW) and tourmaline water (TMW), reconstituted commercially available (Tropicana) and freshly squeezed concentrated juices and the effect of functional waters on physicochemical parameters and sensory evaluation of reconstituted juices was also analyzed. The reconstituted juice exhibited the highest antioxidant activity when the electrolysis current of AlEW was 8A or the tourmaline stone treatment temperature of TMW was 75 °C. Compared with the control group (69.4%), SOD activity of the reconstituted orange juice in the 8A-AlEW (77.2%) and 75 °C-TMW (84.5%) groups increased by 7.8 and 15.1%, respectively. Furthermore, the color and pH of the functional water reconstituted juice were not significantly different from the juice before concentration, and the taste was better. In summary, functional water could enhance the antioxidant activity of concentrated juice as a formula which could provide novel ideas for the development of functional beverages with antioxidant properties.

## 1. Introduction

In 1993, the Japanese Society for Functional Water was founded, and the definition of functional water was proposed. Functional water is an aqueous solution, in which useful functions with reproducibility capability were incorporated in artificial treatment, including electrolyzed water (alkaline electrolyzed water and acidic electrolyzed water), tourmaline water (TMW) [1]. Alkaline electrolyzed water (AlEW), also called electrolyzed reduced water or alkaline ion water, is produced at the cathode by electrolysis [2]. Tourmaline is a natural borosilicate mineral with a complex structure that can release negative ions and generate an electric field on its surface [3,4]. Water flowing through tourmaline stones generates TMW. Both AlEW and TMW are types of functional water with electrochemical properties. In 1997, Shirahata et al. [5] proposed for the first time that AlEW had a superoxide dismutase (SOD)-like effect and could scavenge reactive oxygen species (ROS). Hanaoka [6] demonstrated by electron spin resonance (ESR) that although antioxidants, such as L-ascorbic acid, d-catechin, and quercetin exhibited SOD activity, AlEW increased the SOD activity of these antioxidants. Furthermore, a simpler method, the SOD assay kit-WST, was used in our previous study, and it was indicated that AlEW and TMW enhanced the SOD activity of ascorbic acid [7]. At present, consumption of AlEW is common worldwide. In 2005, the Japanese Ministry of Health, Labor and Welfare approved the AlEW produced by domestic electrolysis devices for daily drinking and medical treatment [8]. It can not only treat gastrointestinal diseases, but also reduce the incidence of diabetes, cardiovascular and cerebrovascular diseases and cancer, and reduce the occurrence of inflammation [9,10,11]. Using this functional water would be a new way to develop functional beverages with antioxidant properties.

Free radicals are known to affect human health and cause cancer, hypertension, and cardiovascular and cerebrovascular diseases [12]. Fruits are rich sources of vitamins, organic acids, minerals, carotenoids, phenolic compounds, and antioxidants that can scavenge free radicals, such as ROS [13]. Regular consumption of fruits and vegetables is associated with a healthier lifestyle and a lower risk of chronic diseases, such as cardiovascular diseases. Hence, for people who live a fast-paced urban lifestyle, fruit juice is one way to easily exogenous antioxidants. Therefore, it is imperative to improve the antioxidant activity of juices. Several studies have proposed methods for improving the antioxidant properties of juice, but most of them are based on processing techniques, such as lactic acid fermentation [14] and reverse osmosis technology [15], to increase the content of antioxidants in juice to enhance its antioxidant properties. However, most juices in fast-food restaurants are reconstituted juices. The main reason for this is the lower distribution cost. Concentrated beverages are usually diluted with purified water and served to customers. During secondary processing, juice should also be enhanced for antioxidant properties. In the present study, a processed concentrated juice was utilized as the raw material, and its antioxidant content was constant. The concentrated juice was diluted with pure water (control), AlEW, and TMW by simulating the preparation of concentrated reconstituted juice in fast food restaurants. The SOD assay kit-WST method was used to investigate the effect of functional water on the antioxidant activity of the reconstituted juices. Moreover, the impact of functional water on the physicochemical properties as well as the sensory quality of the reconstituted juices was also evaluated.

## 2. Materials and Methods

### 2.1. Preparation of Sample Water and Measurement of Physical and Chemical Parameters

Pure water was generated using a pure water generator (Elix Essential UV3, Merck Millipore, Germany), and electrical conductivity (EC) values were measured before use to ensure that the value was 2.0 µS/cm or less. AlEW was generated using an electrolyzed water generator (ROX-20TA, Hoshizaki Corporation, Kagoshima University, Japan; with some modifications by our laboratory) at 6, 8, 10, and 12 A and 10 V. For obtaining TMW, 120 g of Brazilian tourmaline stone was heated in a water bath at 50, 75, and 100 °C for 30 min and cooled to room temperature. Tourmaline stone was placed in a tube of a tourmaline water generator (manufactured by Kagoshima University), and 10 L of pure water was introduced and circulated for 30 min with the power of a pump (A picture of the TMW generator is shown in Supplementary Material, Figure S1.). After the sample water was prepared, the pH, oxidation-reduction potential (ORP), EC, dissolved oxygen (DO), and dissolved hydrogen (DH) were measured using a pH meter (TPX-999, Toko Chemical Laboratory Co., Ltd., Tokyo, Japan), an ORP meter (silver-silver chloride electrode: TRX-90, Toko Chemical Laboratory Co., Ltd., Tokyo, Japan), an EC meter (CD-6021A, CUSTOM Co., Ltd., Tokyo, Japan), a DO meter (SG9-ELK, Mettler-Toledo Co., Ltd., Tokyo, Japan), and a DH meter (ENH-2000, TRUSTLEX Co., Ltd., Osaka, Japan), respectively. The physical and chemical parameters of sample water used in this study are shown in Table 1.

### 2.2. Preparation of Concentrated and Renconstituted Juice

Apple juice and orange juice (100%, Tropicana, Kirin Beverage Co., Ltd., Tokyo, Japan and Tropicana Products, Inc., Harrison, NY, USA) and fresh apples and oranges without signs of mildew, pests, or diseases, were purchased (Nishimuta supermarket in Kagoshima City, Japan). The fresh fruits were washed, the orange peel was removed, the oranges and apples were cut into pieces, and the juice was squeezed using a juicer (MJ-H200, Panasonic, Tokyo, Japan). Filtered (100-mesh sieve) Tropicana and freshly squeezed juice (hereafter collectively referred to as “juice”) were used to remove impurities such as pulp. The physical properties of the original juice were first measured, and then the juice was concentrated. In this experiment, the concentrated juice was prepared according to the freeze concentration method of previous report with some modifications [16]. In brief, the juice was frozen in a refrigerator (Hoshizaki Co., Ltd., Aichi, Japan) at −20 ± 5 °C for 10 h, thawed in a refrigerator at 4 ± 2 °C for 2 h, and the obtained concentrated juice was collected.

Subsequently, the concentrated reconstituted juice was prepared simulating a fast food restaurant. Juice was reconstituted with each sample water (pure water, AlEW, and TMW) to obtain the °Brix value before concentration and refrigerated for later testing.

### 2.3. pH and Total Soluble Solids (TSS) of Juice

The pH of juice (as prepared in Section 2.2) was measured at 25 ± 2 °C using a glass electrode pH meter. The °Brix value of fruit juice was defined as its TSS value, which was measured using a refractometer (IPR-α, AS ONE Co., Ltd., Osaka, Japan) at 25 ± 2 °C. Three replicate experiments were conducted for each set of measurements.

### 2.4. Measurement of the SOD Activity of Juice with SOD Assay Kit-WST

WST is a highly water-soluble tetrazolium salt that can be reduced by superoxide anion (O_2_^−^) to produce a yellow aqueous solution of WST-1 formazan [17]. Compared with the same type of kit, the WST-1 assay was used to determine superoxide radical scavenging activity, whereas the DPPH and the ABTS assays were used to determine the radical scavenging capacity. Since functional water exhibited SOD-like activity, the SOD Assay Kit-WST (Dojinndo Co., Ltd., Kumamoto, Japan) was used as an indicator of antioxidant activity to investigate the effect of functional water on the antioxidant activity of concentrated reconstituted juice.

A 96-well-plate and microplate reader (MPR-A100, AS ONE Co., Ltd., Osaka, Japan) were used for detection. The working solutions were first prepared by diluting 1 mL of WST solution with 19 mL of buffer solution to prepare the WST working solution; the enzyme working solution was prepared by diluting 15 μL of enzyme solution with 2.5 mL of dilution buffer solution. The detailed method and calculated equation (1) are as follows, where the test solution refers to different concentrated reconstituted juices:

(1) Twenty microliters of the test solution was added to the sample and Blank 2 well, and 20 μL of pure water (as control) was added to each Blank 1 and Blank 3 well.
(2) Next, 200 μL of WST working solution was added to each well and mixed thoroughly by using a plate mixer.
(3) Then, 20 μL of dilution buffer was added to each Blank 2 and 3 well.
(4) Subsequently, 20 μL of enzyme working solution was added to the well containing the sample solution and the well of blank 1.
(5) The plate was incubated at 37 °C for 20 min.
(6) The absorbance was measured at 450 nm using a microplate reader and the absorbance value for each well was recorded (e.g., *A _sample_* or *A _blank_*_1_).
(7) The *SOD Activity* was calculated as follows in Equation (1).

(1) $$SOD\;Activity\;\%=\frac{\left[\left(A_{blank1}-A_{blank3}\right)-\left(A_{sample}-A_{blank2}\right)\right]}{\left(A_{blank1}-A_{blank3}\right)}\times 100$$

#### 2.4.1. The Effect of AlEW with Different Electrolysis Currents on the Antioxidant Activity of Juice

AlEW with different electrolytic currents (6, 8, 10, and 12 A) was mixed with 100% Tropicana juice in a 1:1 volume ratio to prepare 50% orange and apple juices. The effect of different electrolytic currents on the antioxidant activity of the juices was measured according to the method described in Section 2.4.

#### 2.4.2. The Effect of TMW on the Antioxidant Activity of Juice at Different Tourmaline Stone Treatment Temperatures

TMW generated with tourmaline stone at different treatment temperatures (50, 75, and 100 °C) was mixed 1:1 by volume with 100% Tropicana juice to prepare 50% orange and apple juice. The effect of different tourmaline stone treatment temperatures on the antioxidant properties of the juices was measured according to the method described in Section 2.4.

#### 2.4.3. The Effect of Functional Water on the Antioxidant Activity of Concentrated Reconstituted Juice

The concentrated reconstituted juice was prepared with functional water according to the optimal conditions obtained in Section 2.4.1 and Section 2.4.2, and its SOD activity was measured.

### 2.5. Color Analysis

The color of the concentrated reconstituted juice was measured using a color reader (CR-13, Konica Minolta, Inc., Tokyo, Japan) equipped with a CLELAB system. Measurements were recorded for “a*” (greenness to redness, positive = red), “b*” (blueness to yellowness, positive = yellow), and “L*” (lightness) [18]. The instrument was calibrated using a standard whiteboard. Average color values were obtained from triplicate measurements.

The total color difference (ΔE) of the sample is calculated by the following Equation (2).

(2) $$\Delta E=\sqrt{{\left(L^{*}-L_{0}^{*}\right)}^{2}+{\left(a^{*}-a_{0}^{*}\right)}^{2}+{\left(b^{*}-b_{0}^{*}\right)}^{2}}$$

In Equation (1), L*, a* and b* are the chromaticity parameters of the sample, and L_0_ *, a_0_ * and b_0_ * are the chromaticity parameters of the unconcentrated juice [19]. According to the value of ΔE, the color difference can be divided into not noticeable (0–0.5), slightly noticeable (0.5–1.5), noticeable (1.5–3.0), well visible (3.0–6.0), and great (6.0–12.0) [20].

### 2.6. Sensory Evaluation

Sensory evaluation of the concentrated juice was performed according to the International Fruit and Vegetable Juice Association (IFU) analysis method (No. 25, 2005) [21] with some modifications. The sensory panelists were trained food science-related scholars from Kagoshima University. Before sensory evaluation, panelists were asked to taste and rank four types of water: sour, sweet, salty, and umami. Finally, 15 members (eight males and seven females) aged between 20 and 70 were selected to form a sensory evaluation panel. Briefly, one set of sensory evaluations consisted of eight cups of juice, reconstituted by PW, AlEW, and TMW, as well as unconcentrated Tropicana and freshly squeezed juice, with three replications for each group. Due to the large number of sample juices the test was divided into two sessions, first for orange and then for apple juice. The test room was ventilated and bright, with the same cup measurements and volume of samples, and the temperature was maintained at 25 °C. Water was provided to rinse the mouth between evaluations. The evaluation results were summarized for comparison and analysis. Grading (1–4, where 1 = minimum, 4 = maximum value) was carried out for the following sensory parameters: color (purity, browning, and typicality), odor (purity, fruity, and intensity), and taste (harmonious, mouth feel, and overall sensation). The definitions of these sensory parameters were explained in detail in the Supplementary Material (Table S1).

### 2.7. Statistical Analysis

Each measurement was performed three times. The results were analyzed by Duncan’s multiple range test using SPSS software (Statistical Package for the Social Sciences; SPSS, Inc., Chicago, IL, USA). Statistical significance was set at *p* < 0.05.

## 3. Results and Discussion

### 3.1. pH and °Brix (TSS) of Juice

**Figure 1:**
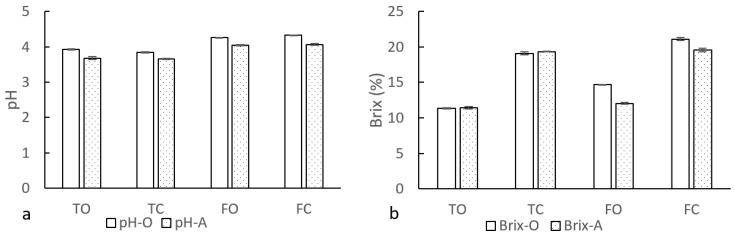
The pH (**a**) and TSS (**b**) of juice. TO, TC, FO and FC are Tropicana original juice, Tropicana concentrated juice, freshly squeezed original juice and freshly squeezed concentrated juice respectively. O and A are the abbreviations for orange and apple. Each treatment was repeated three times and data were expressed by mean ± standard deviation.

To determine the amount of sample water added to the concentrated reconstituted juice, the pH and °Brix of juice were measured before and after concentration (Figure 1). The pH of freshly squeezed juice was higher than that of Tropicana juice. Compared with the original juice, there was no significant change in pH values after concentrated (Figure 1a). Moreover, the °Brix of Tropicana orange and apple juices were both at 11.4%, and the °Brix of concentrated Tropicana juices were 19.1% and 19.4%, respectively. The °Brix values of freshly squeezed apple and orange juices were 14.7% and 12.1%, and the °Brix of concentrated juices were 21.1% and 19.6%, respectively (Figure 1b). Since the pH of juice was no significant change before and after concentrated, the amount of PW was added to the concentrated juice to dilute it to the concentration of the original juice was used as the reconstituted standard. Tropicana orange and apple juices both had the same PW addition amount of 70% (volume fraction of pure water and concentrated juice). The amount of PW addition in the freshly squeezed orange and apple juices were 40% and 60%, respectively. To ensure uniformity in concentration of the concentrated reconstituted juice, the amount of AlEW and TMW added was the same as that of PW. The relationship between the volume of sample water added and the °Brix of the concentrated reconstituted juice is shown in the Supplementary Material (Figure S2), and the type of sample water had no significant effect on the °Brix of the concentrated reconstituted juice.

### 3.2. The Effect of AlEW with Different Electrolysis Currents on the Antioxidant Activity of Juice

**Figure 2:**
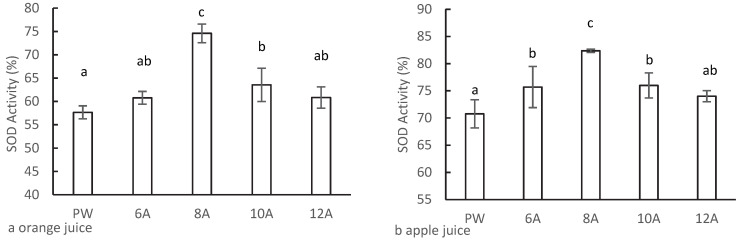
The effect of electrolysis current on SOD activity of AlEW 50% Tropicana juice; (**a**) orange juice; (**b**) apple juice. PW is pure water (control); 6~12A is the electrolysis current of AlEW. The different letters indicated significant difference (*p* < 0.05).

Previous reports have indicated that AlEW can scavenge superoxide anion [22,23], but there is limited information known on the effect of electrolytic conditions on the antioxidant properties of AlEW. In the present study, the effects of different electrolysis conditions of AlEW on the antioxidant activity of 50% Tropicana juice were compared. As shown in Figure 2a, the SOD activity of orange juice, which was diluted with AlEW, showed an initial increasing and then decreasing trend with increasing current intensity that reached a peak at 8 A. The SOD activity of the AlEW group was significantly higher than that of the control group (57.7%). Compared with that of the control group (PW group), the SOD activity at 8 A (74.7%) and 10 A (63.7%) increased by 17.0% and 6.0%, respectively. Additionally, although the SOD activities at 6 A and 12 A were higher than those of the control group (increased by 3.1% and 3.2%, respectively), they were not significantly different from that of the control group. Similar results were obtained with apple juice, where the SOD activity was 4.9, 11.3, and 5.2% higher than that of the control group at 6, 8, and 10 A, respectively (Figure 2b). However, there was no significant difference between 12A and control group.

The results demonstrated that AlEW could enhance the antioxidant activity of antioxidants, which is in agreement with a previous report [23]. In AlEW produced with several different electrolysis currents, AlEW with an electrolysis current of 8 A had an optimal enhancing effect on the antioxidant activity of antioxidant substances. Moreover, excessive current intensity weakened the enhancement effect. Therefore, for subsequent experiments, AlEW with an electrolysis current of 8 A was used to prepare the concentrated reconstituted juice.

### 3.3. Effect of TMW on the Antioxidant Activity of Juice at Different Tourmaline Stone Treatment Temperatures

**Figure 3:**
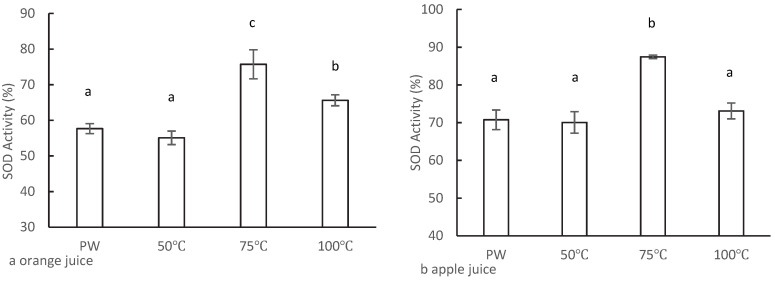
The effect of TMW with different tourmaline stone treatment temperature on SOD activity of 50% Tropicana juice; (**a**) orange juice; (**b**) apple juice. PW is pure water (control); 50~100 °C is the treatment temperature of tourmaline stone. The different letters indicated significant difference (*p* < 0.05).

The most important feature of tourmaline stones is that they possess spontaneous and permanent poles [24]. When the surrounding environment, temperatures or pressure changes, electrons in the tourmaline lattice are transferred, resulting in one end of the tourmaline being positively charged and the other end negatively charged [25]. The effect of TMW produced by the tourmaline stone at different temperatures on the antioxidant activity of the juice was analyzed, and the results are shown in Figure 3. The volume ratio of TMW to 100% Tropicana juice was 1:1 to prepare 50% orange and apple juice. In the treatment temperature range of 50 to 100 °C, the SOD activity of orange juice gradually increased in the order of 50 °C, 100 °C, and 75 °C (Figure 3a). Furthermore, the SOD activity at 75 °C and 100 °C was significantly higher than that in the control group (57.7%), increasing by 18.1% and 8.0%, respectively, while the difference between the 50 °C (55.1%) and the control group was not significant. As shown in Figure 3b, similar results were obtained for apple juice, in which the SOD activity at 75 °C reached a peak of 87.5%, which was significantly higher than that of the control group (70.8%). However, when the treatment temperature was 50 °C and 100 °C, TMW had no enhancement effect on the antioxidant activity of apple juice. It was indicated that TMW could enhance the antioxidant activity of the antioxidant substances in juice.

Studies have been conducted in Japan on the free radical-scavenging active ingredients of AlEW since the 1990s, and they have confirmed that it is the dissolved dihydrogen produced by electrolysis that is the active ingredient of AlEW [8]. Furthermore, TMW could also improve the activity of SOD, and both TMW and AlEW were weakly alkaline (Table 1) and carried electrical energy. The difference is that AlEW contained DH, the ORP value of AlEW was negative, the DH value of TMW was 0, and the ORP value was higher than that of AlEW. Therefore, it is hypothesized that TMW enhances SOD activity because the tourmaline stone was heat-treated and that the electrons within moved and produced electrodes. Water flowed through the tourmaline stone to generate electricity, thereby enhancing the antioxidant ability of the antioxidant substances in the juice.

In addition to the hypothesis that active hydrogen scavenges free radicals, Hanaoka [23] proposed a dissociation hypothesis that electrolysis can enhance the dissociation energy of AlEW and promote dissociation. When the antioxidant substance is dissolved in AlEW, dissociation activity increases, resulting in an increase in antioxidant activity [7]. Therefore, the mechanism involved in TMW induced enhancement of antioxidant properties might be similar to the conjecture of Hanaoka et al., that TMW enhances the dissociation activity of antioxidant substances.

These results indicated that the functional water produced by electrical treatment or electrolysis enhanced the antioxidant activity of antioxidants, but an excessively high treatment temperature or high electrolysis current hindered the enhancement effect of its antioxidant activity. Therefore, TMW with a tourmaline stone treatment temperature at 75 °C was used to prepare concentrated reconstituted juice for subsequent experiments.

### 3.4. Effect of Functional Water on the Antioxidant Activity of Concentrated Reconstituted Juice

**Figure 4:**
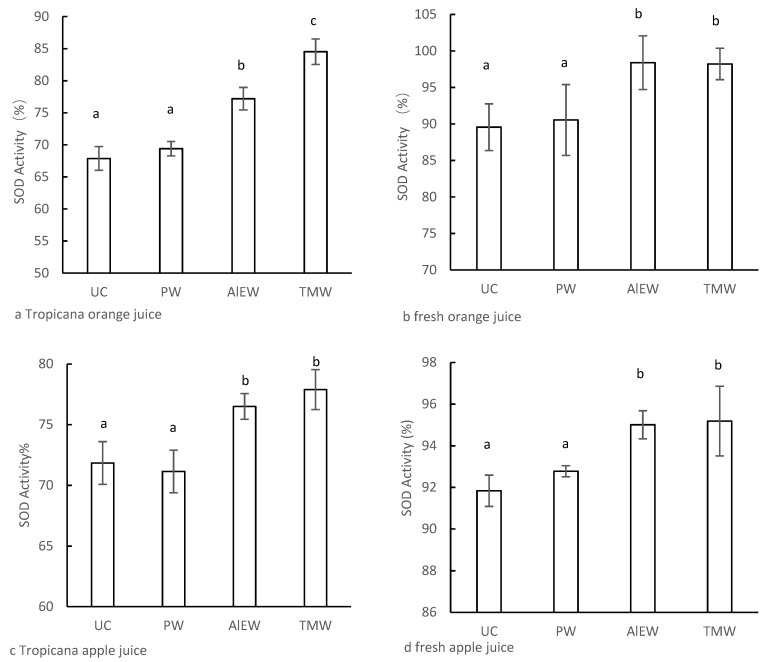
The effect of AlEW and TMW on SOD activity of concentrated reconstituted juice; (**a**) Tropicana orange juice; (**b**) freshly squeezed orange juice; (**c**) Tropicana apple juice; (**d**) Freshly squeezed apple juice. UC is unconcentrated juice; PW is pure water; AlEW is the alkaline electrolyzed water with an electrolysis current of 8 A; TMW is the tourmaline water produced when the treatment temperature of tourmaline stone is 75 °C. The different letters indicated significant difference (*p* < 0.05).

The optimal processing conditions for AlEW and TMW, which could effectively enhance SOD activity, were determined to reconstitute the concentrated juice with 8 A-AlEW or 75 °C-TMW. The effect of functional water on the antioxidant activity of the concentrated reconstituted juice was analyzed, and the results are shown in Figure 4. There was no significant difference in antioxidant activity between reconstituted with pure water (control group) and juice before concentration. As shown in Figure 4a, the SOD activity of 100% Tropicana concentrated reconstituted orange juice of the AlEW and TMW groups were 77.2 and 84.5%, respectively, which were significantly higher than that of the control group (69.4%). For the freshly squeezed orange juice, the SOD activity was increased by 7.9% and 7.7% in the AlEW and TMW groups, respectively (Figure 4b), compared with the control group (*p* < 0.05). However, there was no significant difference in SOD activity between AlEW and TMW. Similar results were obtained in the analysis of the SOD activity of apple juice. Regardless of whether the apple juice was Tropicana or freshly squeezed, the SOD activity of the AlEW and TMW groups was significantly higher than that of the control group (*p* < 0.05). SOD activity increased by 5.4% in AlEW (Figure 4c), 6.7% in TMW (Figure 4c) of Tropicana apple juice, and 2.2% in AlEW (Figure 4d), 2.4% in TMW (Figure 4d) of freshly squeezed apple juice, respectively.

In the present study, AlEW and TMW enhanced the antioxidant activity of concentrated reconstituted juice. Therefore, when concentrated juice is consumed, it might be better to dilute the concentrated fruit juice with AlEW or TMW. Our finding also indicates that these two types of functional waters is a promising option in food processing. From a long-term perspective, concentrated fruit juice is improved when modified with functional water to enhance the antioxidant activity of beverages. It is beneficial to human health, reduces transportation and processing costs of raw materials, and is environmentally friendly. In addition, it is a new type of product with beneficial qualities that attracts consumers who frequent restaurants, fast-food restaurants, and convenience stores. However, the effect of the two types of functional water on the storage properties of juices requires further research.

### 3.5. Effect of Functional Water on the Color and pH of Concentrated Reconstituted Juice

Color is the initial factor perceived by consumers and is considered as one of the main attributes of a beverage, because it might cause the product to be approved or rejected directly [26]. Therefore when developing a new type of beverage, we must first determine the acceptability of its color. The effects of different types of sample waters on the color of the juice are shown in Table 2a,b. The different types of sample waters had no significant effect on the L* and a* values of reconstituted juice. The b* values of AlEW Tropicana reconstituted orange juice were significantly lower than those of the unconcentrated juice (UC), PW, and TMW group. In contrast, a significant difference was not found among the groups for freshly squeezed orange juice. However, the b* value of apple juice of the AlEW group was significantly higher than that of the apple juice of the TMW and was not significantly different from the UC group.

Furthermore, the total color difference (ΔE) of the reconstituted and unconcentrated juice was compared (Table 2). The values of ΔE for each group of reconstituted juice showed no significant differences (*p* < 0.05). Except for the freshly squeezed orange juice, the ΔE values were in the “slightly noticeable” range. The ΔE of PW and TMW freshly squeezed reconstituted orange juice were 0.44 ± 0.9 and 0.42 ± 0.37 (ΔE < 0.5), respectively, while that of AlEW was greater than 0.5 in the “ slightly noticeable” range. Nevertheless, the difference among their three groups was not significant. The results indicated that there was no obvious color difference between functional water reconstituted and unconcentrated juice.

pH is also one of the main properties of fruit juices. The pH values of commercially available 100% orange and apple juice is 3.35–4.2 [27]. The pH value of the juices in this study ranged 3.6–4.3 (Table 2a,b), and different sample water had no effect on the pH value of the juice.

### 3.6. Sensory Evaluation of Concentrated Reconstituted Juice

Sensory evaluation provides a more direct method for evaluating beverage quality [28]. The color, odor, and taste of the concentrated reconstituted juice in this study were analyzed (Table 3a,b). The sensory parameter scores were almost evenly distributed between 2.0 and 3.0, indicating that the juice had a normal fruity color without browning, a pure fruity odor, and moderate sweetness and sourness, which was acceptable. In the orange juice group, there were no significant differences with respect to color or odor. The taste scores of Tropicana juice diluted with AlEW and TMW were significantly higher than those of the control and unconcentrated group; however, there was no significant difference between juices diluted with AlEW and TMW. The taste scores of freshly squeezed juices diluted with TMW and AlEW were higher than the control group but not significantly different from the unconcentrated juice; similar results were observed in the apple juice group. However, freshly squeezed juice reconstituted with TMW had a significantly higher odor score than the control group. Additionally, evaluation panel members stated that the juices in the AlEW and TMW groups were significantly sweeter than those in the control group, with more harmonious sensation in the mouth. Only one individual indicated that there was no difference among the three juices. In summary, AlEW and TMW had a significant effect on the taste of the concentrated reconstituted juice than that of the control group; however, they did not affect the color and odor.

## 4. Conclusions

In this study, we demonstrated that functional water could enhance the antioxidant properties of fruit juice. The SOD activity of juice reached the maximum value when the electrolysis current of AlEW was 8 A or tourmaline stone treatment temperature of TMW was 75 °C, which were determined as the reconstitution conditions of the concentrated juice. The results also showed that the SOD activity of 100% Tropicana concentrated reconstituted orange juice of the AlEW (77.2%) and TMW (84.5%) groups increased by 7.8 and 15.1%, respectively, compared with that of the control group (69.4%). The SOD activity of freshly squeezed orange juice increased by 7.9% and 7.7%, respectively. Tropicana apple juice and freshly squeezed apple juice SOD activity increased by more than 5.4% and 2.2%, respectively. In addition, no significant color difference was observed between the concentrated reconstituted juice of functional water and unconcentrated group. However, functional water could improve the taste of juices. In summary, AlEW and TMW enhanced the antioxidant activity of the antioxidants in the concentrated juice. As a formula for preparation of beverages, the addition of functional water to concentrated fruit juice could provide novel ideas for the development of functional beverages with antioxidant properties.

## Figures and Tables

**Table 1:** The physical and chemical parameters of sample waters ^1^.

	pH	EC (μS/cm)	DO (mg/L)	ORP (mV)	DH (ppm)
PW	6.40 ± 0.17 a	1.0 ± 0.28 a	3.77 ± 0.29 c	305.33 ± 4.15 f	-
AlEW-6A	10.06 ± 0.01 c	789.5 ± 4.95 d	2.90 ± 0.71 b	−245.50 ± 2.12 c	0.56 ± 0.01 a
AlEW-8A	10.25 ± 0.00 d	998.5 ± 0.71 e	2.60 ± 0.42 b	−262.67 ± 2.72 b	0.61 ± 0.00 b
AlEW-10A	10.75 ± 0.00 e	1312.0 ± 2.83 f	2.25 ± 0.21 a	−269.00 ± 4.24 b	0.63 ± 0.01 b
AlEW-12A	11.10 ± 0.00 f	1738.5 ± 2.21 g	2.20 ± 0.14 a	−279.50 ± 6.36 a	0.66 ± 0.02 c
TMW-50 °C	7.58 ± 0.19 b	108.2 ± 6.15 b	2.65 ± 0.21 b	256.67 ± 3.51 d	-
TMW-75 °C	7.68 ± 0.04 b	129.5 ± 5.80 c	3.80 ± 0.87 c	266.00 ± 2.83 d	-
TMW-100 °C	7.76 ± 0.01 b	130.3 ± 2.76 c	3.40 ± 0.14 b	272.50 ± 0.71 e	-

^1^ PW is pure water; AlEW-6,8,10,12A is the electrolysis current of the AlEW; TMW is tourmaline water; TMW-50, 75, 100 °C is the treatment temperature of tourmaline stone; EC is electrical conductance; DO is dissolved oxygen concentration; DH is dissolved hydrogen concentration; ORP is Oxidation-Reduction Potential; “-” means not detection. The different letters indicated significant difference (*p* < 0.05). Each value is expressed as the mean value ± standard deviation of three replicates.

**Table 2:** The physical properties of orange juice ^1^.

**a**					
	L*	a*	b*	**ΔE**	**pH**
O-UC-T	38.67 ± 0.15 a	−1.73 ± 0.25 a	8.70 ± 0.47 b	-	3.92 ± 0.02 b
O-PW-T	38.40 ± 1.04 a	−2.00 ± 0.53 a	9.57 ± 0.98 a	1.22 ± 0.67 a	3.88 ± 0.00 b
O-AlEW-T	38.90 ± 0.43 a	−1.50 ± 0.64 a	7.17 ± 0.23 c	1.47 ± 0.36 a	3.89 ± 0.01 b
O-TMW-T	38.83 ± 0.41 a	−1.50 ± 0.30 a	9.90 ± 0.85 a	1.36 ± 0.42 a	3.88 ± 0.01 b
O-UC-F	14.27 ± 0.28 b	−1.40 ± 0.20 a	0.19 ± 0.08 d	-	4.26 ± 0.01 b
O-PW-F	14.03 ± 0.21 b	−1.57 ± 0.25 a	0.23 ± 0.21 d	0.44 ± 0.39 b	4.28 ± 0.01 a
O-AlEW-F	13.60 ± 0.10 b	−1.43 ± 0.15 a	−0.03 ± 0.16 d	0.83 ± 0.20 ab	4.32 ± 0.01 a
O-TWM-F	14.13 ± 0.06 b	−1.17 ± 0.35 a	0.00 ± 0.2 d	0.42 ± 0.37 b	4.30 ± 0.01 a
**b**					
	L*	a*	b*	**ΔE**	**pH**
A-UC-T	29.63 ± 0.81 a	−1.09 ± 0.17 b	2.48 ± 0.33 bc	-	3.68 ± 0.02 b
A-PW-T	28.57 ± 0.12 a	−1.07 ± 0.15 b	2.73 ± 0.40 b	1.21 ± 0.36 a	3.74 ± 0.01 b
A-AlEW-T	30.17 ± 0.64 a	−1.10 ± 0.10 b	2.63 ± 0.23 b	0.76 ± 0.66 a	3.76 ± 0.00 b
A-TMW-T	30.17 ± 0.31 a	−1.10 ± 0.10 b	2.10 ± 0.17 c	1.29 ± 0.19 a	3.74 ± 0.01 b
A-UC-F	26.00 ± 0.36 b	2.07 ± 0.25 a	3.53 ± 0.49 ab	-	4.05 ± 0.03 a
A-PW-F	25.13 ± 0.71 b	2.10 ± 0.52 a	2.70 ± 0.26 b	1.43 ± 0.56 a	4.04 ± 0.01 a
A-AlEW-F	26.50 ± 0.17 b	1.90 ± 0.35 a	4.20 ± 0.35 a	1.35 ± 0.49 a	4.10 ± 0.02 a
A-TMW-F	26.33 ± 0.50 b	2.03 ± 0.65 a	3.17 ± 0.31 b	1.01 ± 0.56 a	4.03 ± 0.01 a

^1^ O is orange juice; A is apple juice; UC is unconcentrated fruit juice; T is the Tropicana juice reconstituted by the sample water; F is the freshly squeezed juice reconstituted by the sample water; “-” means not detection. The different letters indicated significant difference (*p* < 0.05). Each value is expressed as the mean value ± standard deviation of three replicates.

**Table 3:** The sensory evaluation of orange juice ^1^.

**a**			
	**Color**	**Odor**	**Taste**
O-UC-T	2.53 ± 0.96 a	2.67 ± 0.62 a	2.27 ± 0.46 c
O-PW-T	2.40 ± 0.74 a	2.58 ± 0.75 a	2.00 ± 0.64 c
O-AlEW-T	2.73 ± 0.80 a	2.67 ± 0.56 a	2.56 ± 0.54 b
O-TMW-T	2.67 ± 0.82 a	2.53 ± 0.78 a	2.51 ± 0.81 b
O-UC-F	2.60 ± 0.63 a	2.40 ± 0.83 a	2.47 ± 0.83 bc
O-PW-F	2.53 ± 0.88 a	2.22 ± 0.60 a	2.24 ± 0.50 c
O-AlEW-F	2.80 ± 0.86 a	2.40 ± 0.61 a	2.96 ± 0.50 a
O-TWM-F	2.73 ± 0.80 a	2.44 ± 0.70 a	2.64 ± 0.47 ab
**b**			
	**Color**	**Odor**	**Taste**
A-UC-T	2.53 ± 0.74 a	2.40 ± 0.73 ab	2.56 ± 0.63 ab
A-PW-T	2.40 ± 0.74 a	2.27 ± 0.96 ab	2.32 ± 0.55 b
A-AlEW-T	2.93 ± 0.88 a	2.58 ± 0.75 a	2.72 ± 0.43 a
A-TMW-T	2.80 ± 0.68 a	2.44 ± 0.65 ab	2.82 ± 0.51 a
A-UC-F	2.40 ± 0.91 a	2.37 ± 0.72 ab	2.60 ± 0.98 ab
A-PW-F	2.33 ± 0.89 a	1.96 ± 0.65 b	2.48 ± 0.46 b
A-AlEW-F	2.73 ± 0.70 a	2.31 ± 0.50 ab	2.82 ± 0.61 a
A-TWM-F	2.73 ± 0.88 a	2.62 ± 0.90 a	2.91 ± 0.70 a

^1^ O is orange juice; A is apple juice; UC is unconcentrated fruit juice; T is the Tropicana juice reconstituted by the sample water; F is the freshly squeezed juice reconstituted by the sample water. The different letters indicated significant difference (*p* < 0.05). Each value is expressed as the mean value ± standard deviation of three replicates.

## Data Availability

The date are available from the corresponding author.

## Supplementary Materials

The following supporting information can be downloaded at: https://www.mdpi.com/article/10.3390/foods11162531/s1, Figure S1: Tourmaline water (TMW) generator; Figure S2: The relationship between volume of addition of sample water and the °Brix of the (a-tropicana, b-freshly squeezed) concentrated reconstituted juice; Table S1: Sensory parameters scoring instructions.

## Abbreviations

AlEW	alkaline electrolyzed water
TMW	tourmaline water
SOD	superoxide dismutase
PW	pure water
EC	electrical conductance
DO	dissolved oxygen
DH	dissolved hydrogen
ORP	oxidation reduction potential

## References

[1] The Japanese Society for Functional Water. http://www.fwf.or.jp/gakkai.html.

[2] Tanaka Y., Saihara Y., Izumotani K., Nakamura H. (2018). Daily ingestion of alkaline electrolyzed water containing hydrogen influences human health, including gastrointestinal symptoms. Med. Gas. Res..

[3] Jia W., Wang C., Ma C., Wang J., Sun H. (2018). Element uptake and physiological responses of Lactuca sativa upon co-exposures to tourmaline and dissolved humic acids. Environ. Sci. Pollut. Res..

[4] Liang Y., Tang X., Zhu Q., Han J., Wang C. (2021). A review: Application of tourmaline in environmental fields. Chemosphere.

[5] Shirahata S., Kabayama S., Nakano M., Miura T., Kusumoto K., Gotoh M., Hayashi H., Otsubo K., Morisawa S., Katakura Y. (1997). Electrolyzed–Reduced Water Scavenges Active Oxygen Species and Protects DNA from Oxidative Damage. Biochem. Biophys. Res. Commun..

[6] Hanaoka K. (2001). Antioxidant effects of reduced water produced by electrolysis of sodium chloride solutions. J. Appl. Electrochem..

[7] Wu T., Hiroshima M., Tachibana C., Gaja M., Nagahama A., Kamitani Y. (2022). Comparative Study of Alkaline Electrolyzed Water and Tourmaline Water in terms of their Enhancing Effect on the SOD Activity of Ascorbic Acid. J. Funct. Water.

[8] Marc H., Jacques C. (2013). Physico-Chemical, Biological and Therapeutic Characteristics of Electrolyzed Reduced Alkaline Water (ERAW). Water.

[9] Jin D., Ryu S.H., Kim H.W., Yang E.J., Lim S.J., Ryang Y.S., Chung C.H., Park S.K., Lee K.J. (2006). Anti-Diabetic Effect of Alkaline-Reduced Water on OLETF Rats. Biosci. Biotechnol. Biochem..

[10] Ye J., Li Y., Hamasaki T., Nakamichi N., Komatsu T., Kashiwagi T., Teruya K., Nishikawa R., Kawahara T., Osada K. (2008). Inhibitory Effect of Electrolyzed Reduced Water on Tumor Angiogenesis. Biol. Pharm. Bull..

[11] Li Y., Nishimura T., Teruya K., Maki T., Komatsu T., Hamasaki T., Kashiwagi T., Kabayama S., Shim S.-Y., Katakura Y. (2002). Protective mechanism of reduced water against alloxan-induced pancreatic β-cell damage: Scavenging effect against reactive oxygen species. Cytotechnology.

[12] Wang J., Yuan X., Jin Z., Tian Y., Song H. (2007). Free radical and reactive oxygen species scavenging activities of peanut skins extract. Food Chem..

[13] Liu R.H. (2013). Health-Promoting Components of Fruits and Vegetables in the Diet. Adv. Nutr. Int. Rev. J..

[14] Qi J., Huang H., Wang J., Liu N., Chen X., Jiang T., Xu H., Lei H. (2021). Insights into the improvement of bioactive phytochemicals, antioxidant activities and flavor profiles in Chinese wolfberry juice by select lactic acid bacteria. Food Biosci..

[15] Gunathilake K., Yu L., Rupasinghe H. (2004). Reverse osmosis as a potential technique to improve antioxidant properties of fruit juices used for functional beverages. Food Chem..

[16] Yoda T., Miyaki H., Saito T. (2021). Effect of container shape on freeze concentration of apple juice. PLoS ONE.

[17] SOD Assay Kit—WST Technical Manual. https://www.dojindo.co.jp/manual/S311e.pdf.

[18] López N., Pérez L., Carbonell B., García C. (2007). Use of Natural and Modified Cyclodextrins as Inhibiting Agents of Peach Juice Enzymatic Browning. J. Agric. Food Chem..

[19] Rodríguez L.M.N., Arias R., Soteras T., Sancho A., Pesquero N., Rossetti L., Tacca H., Aimaretti N., Cervantes M.L.R., Szerman N. (2021). Comparison of the quality attributes of carrot juice pasteurized by ohmic heating and conventional heat treatment. LWT.

[20] Cserhalmi Z., Sass-Kiss Á., Tóth-Markus M., Lechner N. (2006). Study of pulsed electric field treated citrus juices. Innov. Food Sci. Emerg. Technol..

[21] International Fruit and Vegetable Juice Association Available. https://ifu-fruitjuice.com/.

[22] Lee M.Y., Kim Y.K., Ryoo K.K., Lee Y.B., Park E.J. (2006). Electrolyzed-Reduced Water Protects Against Oxidative Damage to DNA, RNA, and Protein. Appl. Biochem. Biotechnol..

[23] Hanaoka K., Sun D., Lawrence R., Kamitani Y., Fernandes G. (2004). The mechanism of the enhanced antioxidant effects against superoxide anion radicals of reduced water produced by electrolysis. Biophys. Chem..

[24] Luo G., Chen A., Zhu M., Zhao K., Zhang X., Hu S. (2020). Improving the electrocatalytic performance of Pd for formic acid electrooxidation by introducing tourmaline. Electrochimica Acta.

[25] Ma C., Christianson L., Huang X., Christianson R., Cooke R.A., Bhattarai R., Li S. (2020). Efficacy of heated tourmaline in reducing biomass clogging within woodchip bioreactors. Sci. Total Environ..

[26] Martín G., García M., Varo M., Mérida J., Serratosa M. (2021). Phenolic compounds, antioxidant activity and color in the fermen-tation of mixed blueberry and grape juice with different yeasts. LWT Food Sci. Technol..

[27] Braverman J. The pH Levels of Apple, Orange, Grape & Cranberry Fruit Juices. https://healthyeating.sfgate.com/ph-levels-apple-orange-grape-cranberry-fruit-juices-12062.html.

[28] Martins I., Souza C., Alcantara M., Rosenthal A., Ares G., Deliza R. (2022). How are the sensory properties perceived by con-sumers? A case study with pressurized tropical mixed juice. Food Res. Int..

