# Supplementary material for: Effect of Functional Water on the Antioxidant Property of Concentrated Reconstituted Juice
Source: Foods. 2022 Aug 21;11(16):2531. doi: 10.3390/foods11162531 (PMC9407226; doi:10.3390/foods11162531)
Supplement: Supplementary file 1 [file foods-11-02531-s001.zip › foods-1854851-supplementary.pdf]

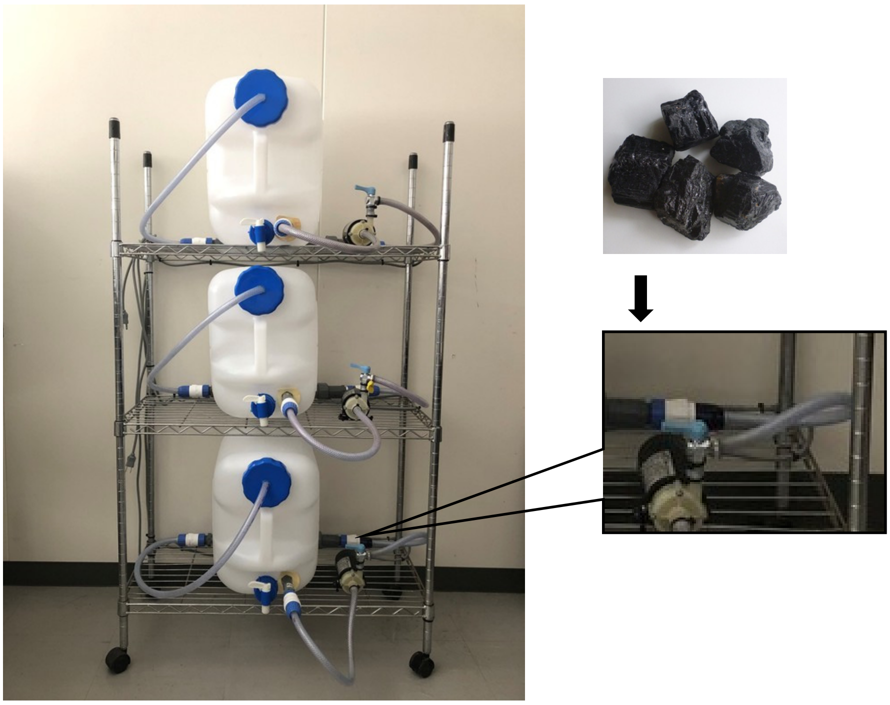

Figure S1 Tourmaline water (TMW) generator

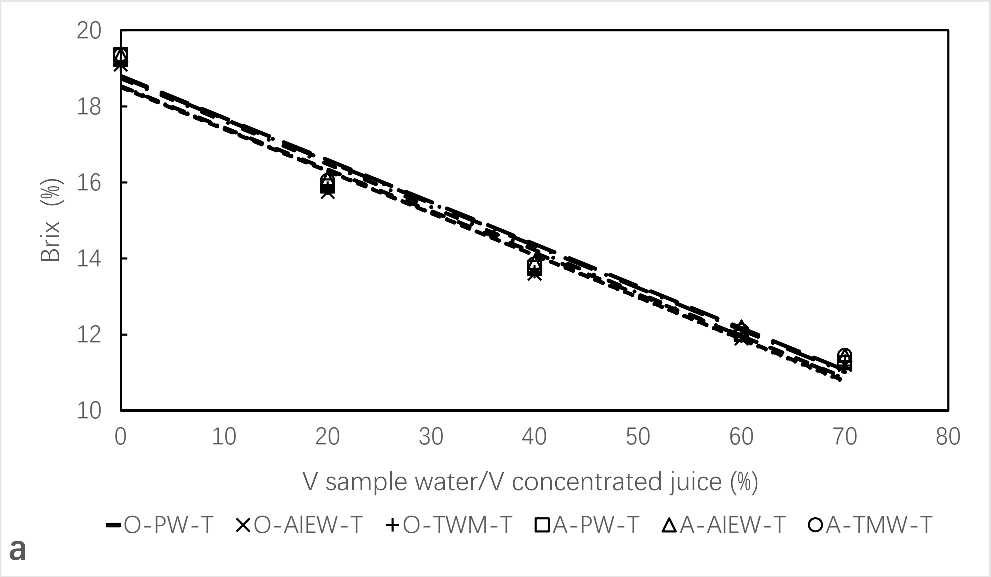

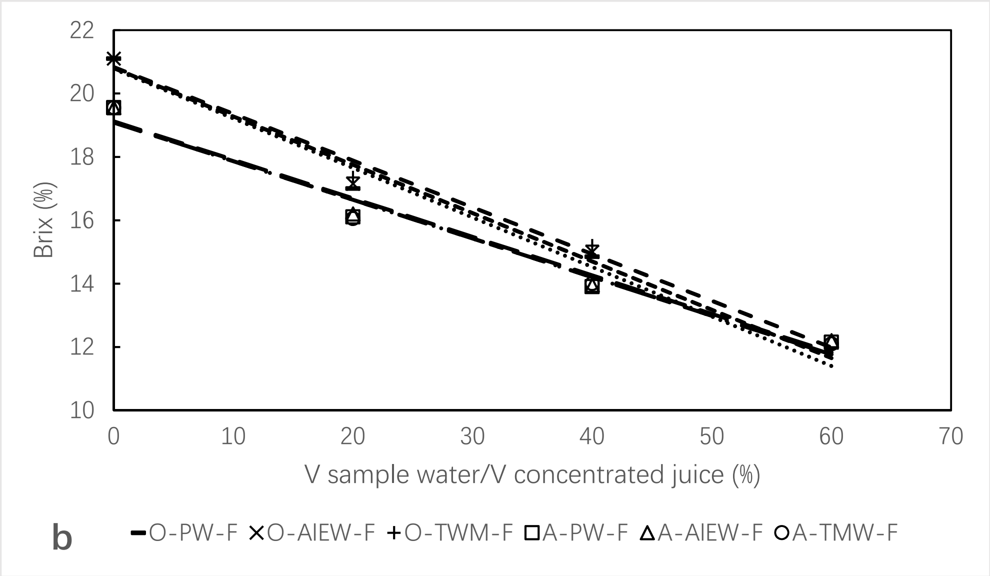

Figure S2 The relationship between volume of addition of sample water and the °Brix of the (a-tropicana, b-freshly squeezed) concentrated reconstituted juice.

a, O is orange; A is apple; T is tropicana juice; F is freshly squeezed juice; PW, AlEW and TMW refer to the concentrated reconstituted juice prepared by this kind of sample water.

Table S1 Sensory parameters scoring instructions [a]

Score	Color	odor	taste
3.0 – 4.0	Typical fresh fruit color without browning and sedimentation	Intense fruity odor	Harmonious pleasant taste
2.0 – 3.0	Normal fresh fruit color with slight sedimentation without browning	Clearly fruity odor	Fruity with moderate sweetness and acidity
1.0 – 2.0	Dark color with a little sedimentation and browning	Lightly fruity odor	Fruity, discordant acidity and sweetness
0.0 – 1.0	Abnormal color with obvious precipitation	No fruity or unpleasant odor	Pungent unpleasant taste

[a] According to the IFU analysis method (No. 25, 2005) with some modifications.
